# Nasal Administration of *Durvillaea antarctica* Fucoidan Inhibits Lung Cancer Growth in Mice Through Immune Activation

**DOI:** 10.3390/ph18091354

**Published:** 2025-09-09

**Authors:** Hee Sung Kim, Peter C. W. Lee, Jun-O Jin

**Affiliations:** 1 Department of General Surgery, Hallym University College of Medicine, Hallym Medical Center, Anyang 14068, Republic of Korea; os-khs@hanmail.net; 2 Department of Biochemistry and Molecular Biology, Brain Korea 21 Project, Asan Medical Center, University of Ulsan College of Medicine, Seoul 05505, Republic of Korea; 3 Department of Microbiology, Brain Korea 21 Project, Asan Medical Center, University of Ulsan College of Medicine, Seoul 05505, Republic of Korea

**Keywords:** fucoidan, *Durvillaea antarctica*, mucosal immune activation, immunotherapy, lung cancer

## Abstract

**Background:** Various studies have demonstrated fucoidan’s immunomodulatory effects. A previous study reported the anticancer effects of *Durvillaea antarctica* fucoidan (DAF) via immune activation in mice. **Methods:** In this study, we confirmed the DAF’s pulmonary immune activation ability by nasal administration of the dendritic cells (DCs) and T cells. Furthermore, we examined its ability to enhance the efficacy of lung cancer treatment by combining it with anti-PD-L1 antibodies to activate the lung immune response. **Results:** Nasal DAF administration increased C-C chemokine receptor type 7 expression in DCs and promoted DC migration to the mediastinal lymph nodes (mLN). Specifically, DAF increased conventional DC type 1 (cDC1) and cDC2 numbers in mLN and potently activated cDC1. Furthermore, the nasal administration of DAF increased the production of inflammatory cytokines in the lungs and peripheral blood. Repeated intranasal administration of DAF induced T-cell activation, resulting in the enhanced production of interferon-gamma and tumor necrosis factor-alpha in CD4 T and CD8 T cells. CD8 T cells also showed increased secretion of cytotoxic mediators after DAF treatment, and the proportion of Tregs expressing FoxP3 decreased in the mLN. DAF inhibited lung cancer growth in Lewis lung carcinoma 2 cells, which was enhanced by combining it with an anti-programmed death-ligand 1 antibody. Finally, the anticancer effects of DAF were not observed in mice with depleted CD4-positive and CD8-positive cells. **Conclusions:** Nasal administration of DAF may inhibit lung cancer growth by inducing lung immune activation and is expected to be helpful as an immune activator for nasal administration.

## 1. Introduction

Natural compounds extracted from plants have been developed as biologically active modulators [1,2,3,4]. In particular, plant-extracted polysaccharides have been reported to possess various immune-regulatory abilities [1,5]. Fucoidan, a primary component of seaweed-derived polysaccharides, has various physiological activities [2,3,6,7,8] including immune activities and anticancer effects [3]. Fucoidan has an immunosuppressive effect when administered orally, making it effective in treating inflammatory diseases [3,7]. Furthermore, intravenous or intraperitoneal administration of fucoidan is effective in cancer immunotherapy because it induces immune activity [6,9,10]. Fucoidan exhibits immune activity when administered through the nasal cavity [11].

Cancer immunotherapy suppresses tumor growth by activating immune cells to attack tumor cells [12,13,14]. Cancer immunotherapy can be derived from the activation of cytotoxic T lymphocytes (CTLs), which exhibit cytotoxicity [12]. Natural killer cells and CTLs induce tumor cell death by secreting pro-inflammatory cytokines such as interferon-gamma (IFN-γ), tumor necrosis factor-alpha (TNF-α), and cytotoxic mediators such as granzyme and perforin [12,13]. The induction of CTL activation is achieved by introducing external substances, such as antibodies, and interactions between cells, such as dendritic cells (DCs) [15,16].

DCs are potent antigen-presenting cells that link innate and adaptive immunity [16]. DCs are activated by recognizing pathogens that have invaded from the outside [16]. Activated dendritic cells have several characteristics. (1) After antigen engulfment, they move to the lymph node (LN), where T cells gather for T cell activation. (2) After moving to the LN, DCs present engulfed antigens to major histocompatibility complex (MHC) classes I and II. (3) DCs upregulate the expression of co-stimulators after antigen uptake in LNs to induce T cell activation and proliferation. (4) Finally, DCs secrete pro-inflammatory cytokines for T-cell differentiation. These factors serve as indicators of DC activity [17].

Research on DC is being conducted more actively in vitro than in vivo; however, substantial differences remain between DCs, making it difficult to apply information on in vitro DC activity to in vivo research [18]. Although isolating DC subtypes in vivo is possible, studying their functions using DCs differentiated in vitro. In particular, in vivo DCs in mice can be separated into two types: conventional DC type 1 (cDC1) and cDC2, depending on the presence or absence of CD8 expression; cDC1 induces CTL activation, while cDC2 promotes the activation of helper T cells [18]. Therefore, the induction of CTL activity by cDC1 is an important indicator in cancer immunotherapy [12].

We previously confirmed the immune-activating ability of fucoidans extracted from *Durvillaea antarctica* [6]. *D. antarctica* fucoidan (DAF) induced splenic DC activity and inhibited tumor growth when administered intraperitoneally [6]. Additionally, the immune-activating ability of DAF enhances the effect of an anti-programmed death-ligand 1 (anti-PD-L1) antibody, which potently inhibits tumor growth [6]. Although the immune activation ability of DAF through intraperitoneal administration has been verified [6,19], a method for treating lung cancer has not been studied. In this study, we examined the immunostimulatory effect of DAF in the mediastinal LN (mLN) by nasal administration and used it to enhance anti-PD-L1 antibody function to determine whether it could inhibit lung cancer growth.

## 2. Results

### 2.1. Nasal Administration of DAF Induced Migration of DCs to the mLN

**Figure 1:**
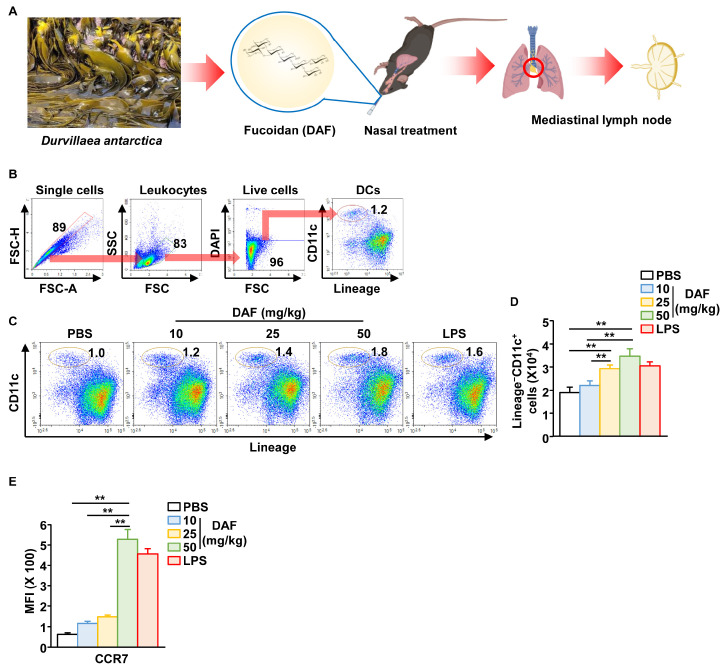
Migration of dendritic cells (DCs) to mediastinal lymph node (mLN) following intranasal administration of *Durvillaea antarctica* fucoidan (DAF). (**A**) Schematic illustration of mLN harvest after intranasal administration of DAF. CD11c^+^Lineage^−^ cells in live leukocytes were referred to as DCs. (**B**) Identification of DC population in mLN using flow cytometry. (**C**) Dose-dependent changes in DC percentage of mLN in DAF. (**D**) Changes in the number of DCs within mLN. (**E**) Mean fluorescence intensity of C-C chemokine receptor type 7 (CCR7) in mLN DCs (right panel). The experiments were repeated twice, with three samples per condition (n = 6), ** *p* < 0.01.

Since intraperitoneal administration of DAF induced splenic DC activation in our previous study [6], we examined DC activation in the mLN following intranasal administration of DAF. As shown in Figure 1A, DAF was administered nasally, and mLN were harvested 18 h after administration. The mLN DC population was identified, as shown in Figure 1B. DAF induced a gradual, dose-dependent increase in the frequency and percentage of DCs in mLN (Figure 1C). The absolute number of DCs in the mLN was also significantly increased by 25 and 50 mg/kg DAF administration compared to that by the phosphate-buffered saline (PBS)-treated control (Figure 1D). In addition, the levels of C-C chemokine receptor type 7, which induces the migration of activated DCs, increased in a dose-dependent manner following DAF treatment (Figure 1E and Figure S1). Lipopolysaccharide (LPS) was used as a positive control, and 50 mg/kg DAF showed a slightly higher DC activation effect than LPS. Therefore, the intranasal administration of DAF may induce the migration of DC into the mLN.

### 2.2. Nasal Administration of DAF Promoted Activation of mLN DCs

**Figure 2:**
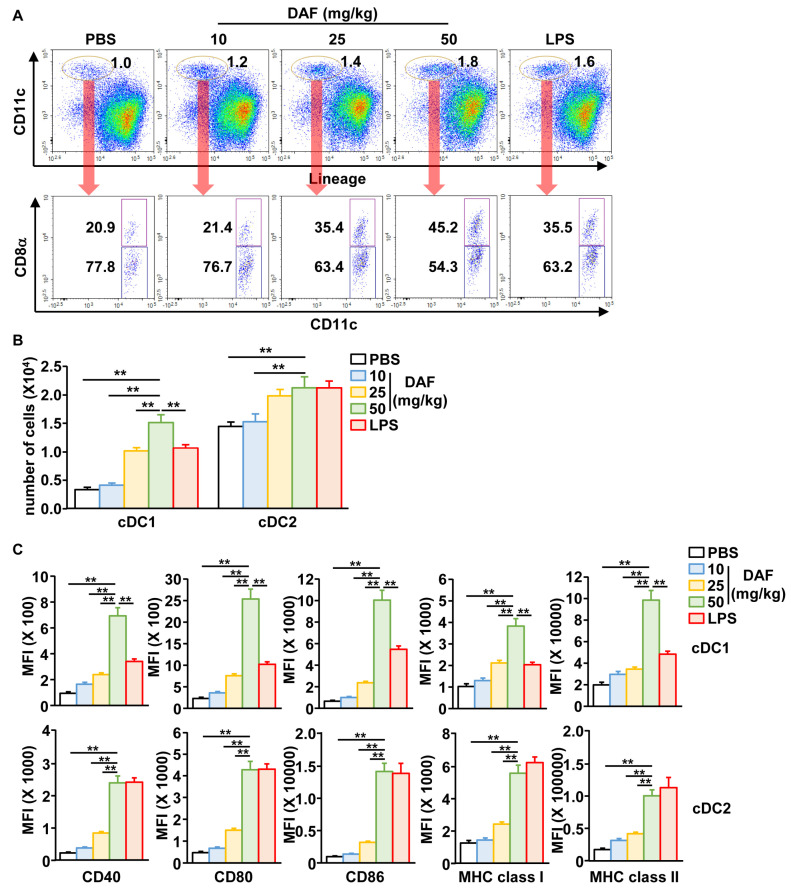
DAF-induced activation of cDC1 and cDC2 in mLN by nasal administration. (**A**) CD11c^+^Lineage^−^ cells in live leukocytes were compartmentalized into CD8α^+^ cDC1 and CD8α^−^ cDC2. (**B**) Absolute number of cDC1 and cDC2 in mLN. (**C**) Mean fluorescence intensity (MFI) of the costimulator and MHC molecules in cDC1 (**upper panel**) and cDC2 (**lower panel**). The experiments were repeated twice, with three samples per condition (n = 6), ** *p* < 0.01.

Next, we examined the activation of mLN DC following nasal administration of DAF. The mLN DCs were divided into CD8-positive cDC1 and CD8-negative cDC2 (Figure 2A). The percentage of CD8-positive cDC1 cells increased dramatically while the number of cDC1 and cDC2 in the mLN gradually increased in a dose-dependent manner after DAF administration (Figure 2A). In particular, cDC1 counts tended to increase more with 50 mg/kg DAF than when the positive control group was administered LPS (Figure 2A). Additionally, the expression of the co-stimulators CD40, CD80, CD86, and MHC classes I and II in both cDC1 and cDC2 was substantially increased by DAF in a dose-dependent manner (Figure 2C and Figure S2). Similar to the increase in cDC1 number induced by DAF, DAF was shown to induce cDC1 activation more potently than LPS. Furthermore, when comparing oral, intraperitoneal, and intranasal administration of DAF, intranasal administration induced DC activation in the mLN. However, it did not demonstrate efficiency in DC activation in the spleen (Figure S3). Intraperitoneal administration of DAF promoted the activation of splenic DCs but not mLN DCs (Figure S3). Oral administration of DAF did not affect the activity of splenic or mLN DCs (Figure S3).

### 2.3. Pro-Inflammatory Cytokine Production by Nasal Administration of DAF in the Lungs and Blood

**Figure 3:**
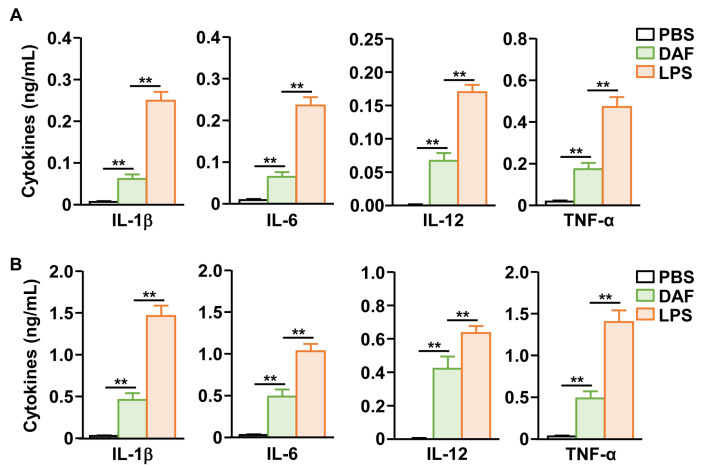
DAF-induced pro-inflammatory cytokine production in bronchoalveolar lavage (BAL) fluid and blood serum. (**A**) DAF (50 mg/kg) was administered to C57BL/6 mice. Indicated pro-inflammatory cytokine concentration in BAL fluid was analyzed 24 h after treatment (DAF). (**B**) The levels of indicated cytokines in the blood serum were analyzed 24 h after nasal administration of DAF. The experiments were repeated twice with three samples per condition (n = 6), ** *p* < 0.01.

The secretion of pro-inflammatory cytokines, another important indicator of DC activity [20], was tested after nasal administration of DAF. DAF significantly increased the secretion of interleukin-1 beta (IL-1β), IL-6, IL-12, and TNF-α in bronchoalveolar lavage (BAL) fluid compared to that of the PBS control group (Figure 3A). Furthermore, we confirmed that the concentration of these pro-inflammatory cytokines in the serum of peripheral blood increased when DAF was administered nasally compared to that in the PBS-treated controls (Figure 3B). However, the secretion levels of these pro-inflammatory cytokines by DAF were significantly lower in both BAL fluid and serum than in the positive control LPS (Figure 3). Therefore, the nasal administration of DAF may induce the production of pro-inflammatory cytokines in the lungs and blood.

### 2.4. DAF Promoted Activation of T Cells in mLN

**Figure 4:**
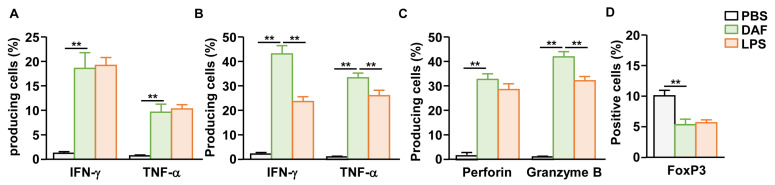
Activation of mLN T cells by nasal administration of DAF. C57BL/6 mice were administered 50 mg/kg DAF intranasally every 3 d. The mLNs of mice were harvested 3 d after the final administration. (**A**) Intracellular levels of IFN-γ and TNF-α in mLN CD4 T cells. Mean producing cells of indicated cytokines. (**B**) IFN-γ and TNF-α producing levels in mLN CD8 T cells. (**C**) Mean positive cells of indicated cytotoxic mediators in mLN CD8 T cells. (**D**) FoxP3-positive cells within the CD4 T cell population in the mLNs. The experiments were repeated twice, with three samples per condition (n = 6), ** *p* < 0.01.

Because nasal administration of DAF activates DCs in the mLN, we next examined whether it could induce T cell activation in the mLN. DAF (50 mg/kg) was administered intranasally twice at 3 d intervals, and the mLN were harvested 3 d after the final administration to confirm T-cell activity. Treatment with DAF induced the intracellular production of IFN-γ and TNF-α in CD4 T cells of mLN, at levels similar to those of the positive control group (LPS) (Figure 4A and Figure S4A). CD8 T cells in mLN also showed a dramatic increase in intracellular production of IFN-γ and TNF-α by DAF (Figure 4B and Figure S4B). Furthermore, in CD8 T cells, perforin and granzyme B, which are cytotoxic mediators that directly induce tumor cell death, were highly expressed by DAF (Figure 4C and Figure S4C). IFN-γ, TNF-α, and granzyme B produced by DAF in the CD8 T cells of mLN were significantly higher than in the positive control, i.e., LPS-induced expression (Figure 4B,C). Moreover, intranasal treatment with DAF reduced the proportion of FoxP3-positive CD4 T cells, representing regulatory T cells, within the mLN (Figure 4D and Figure S4D). Therefore, intranasal administration of DAF may induce T cell activation in the mLN, and CD8 T cells may exhibit stronger immune activity than those stimulated by LPS.

### 2.5. Nasal Administration of DAF Enhanced the Anticancer Effect of Anti-PD-L1 Antibody

**Figure 5:**
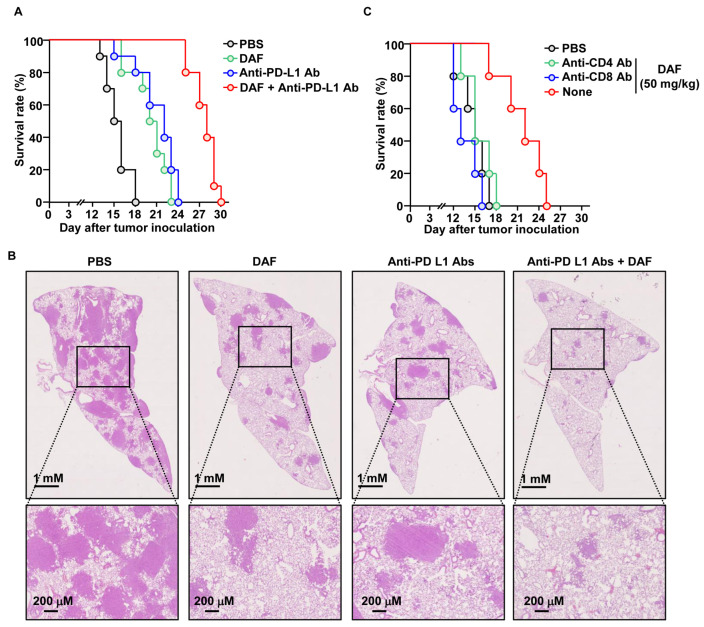
Enhanced anticancer effect of anti-PD-L1 antibody by DAF in lung cancer. C57BL6 mice were intravenously injected with 2 × 10^5^ LL2 cells. Five days after tumor cell injection, DAF (50 mg/kg, nasally) and anti-PD-L1 antibodies (10 mg/kg, intraperitoneally) were administered at 3 d intervals starting 7 d later. (**A**) Survival rate of lung cancer mice treated with DAF and anti-PD-L1 antibodies (n = 10 for each group). (**B**) Histology of the lung on day 15 after tumor injection. (**C**) Seven days after lung cancer cell administration to C57BL6 mice, DAF was administered intranasally at 3 d intervals. During DAF treatment, anti-CD4 or anti-CD8 antibodies were administered at 2 d intervals, and the survival rate of the mice (n = 5 for each group) was monitored.

Based on the DC and T-cell activation abilities of DAF, we examined its adjuvant effects on anti-PD-L1 antibodies. Lung cancer was induced in C57BL6 mice by administering Lewis lung carcinoma 2 (LL2) cells via the tail vein. DAF and anti-PD-L1 antibodies were administered at 3 d intervals starting 7 d later. DAF was administered intranasally at a concentration of 50 mg/kg, and anti-PD-L1 antibody was administered intraperitoneally at a concentration of 10 mg/kg. All mice in the control group administered PBS died 18 d after tumor administration, whereas mice in the control group administered DAF and anti-PD-L1 antibody survived for 23 and 24 d, respectively (Figure 5A). Furthermore, the group administered DAF and anti-PD-L1 antibodies in combination survived for up to 30 d after tumor administration (Figure 5A). The PBS group showed tumor infiltration in many areas 15 d after tumor administration. In contrast, the groups treated with DAF and anti-PD-L1 antibodies showed tumors in relatively few areas (Figure 5B). In the group treated with the combination of DAF and anti-PD-L1 antibodies, the tumor cells infiltrated only small areas (Figure 5B). Furthermore, the inhibitory effect of DAF on lung cancer growth was not observed in mice with depleted CD4-positive or CD8-positive cells (Figure 5C). Therefore, DAF may enhance the anticancer effect of anti-PD-L1 antibodies due to immune activation.

## 3. Discussion

The immunomodulatory properties of fucoidans have been confirmed in several studies [2,6,7,8,11,21]. Fucoidan extracted from *Fucus vesiculosus* (Sigma-Aldrich, St. Louis, MO, USA) has been used in various studies and exhibits immune activity and suppressive abilities [22,23]. However, whether fucoidans activate or suppress immunity remains a matter of controversy [10,24]. Studies on the effects of immune activation and immune suppression have been published, although the same *F. vesiculosus* fucoidan was used [22,23]. Although there are cases where the effects of activation and inhibition differ depending on the route of administration, there are also cases where opposite results are shown, even with the same route of administration, which confuses researchers [24]. According to a previous study, DAF activates immune cells in the spleen when administered intraperitoneally [6]. In this study, DAF was administered intranasally and its effects on pulmonary immune regulation were confirmed. Nasal administration of DAF induced the activation of mLN DCs and T cells and inhibited lung cancer growth. These findings suggested that DAF has immunoactivating capabilities. However, since the immunosuppressive ability is usually studied in inflammatory or infectious diseases and its efficacy is confirmed through oral administration, it is necessary to verify the immunomodulatory ability of DAF under strict experimental conditions.

The efficacy of fucoidans varies greatly depending on the type of natural product being extracted. In previous studies, we compared and analyzed the immunoregulatory properties of different types of fucoidans [3,21,22]. However, the efficacy of fucoidan varies depending on the harvest time and extraction method, even for the same seaweed, making it challenging to determine the reason for the difference in efficacy [10,24]. Changes in activity according to the composition of monosaccharides were confirmed in a study to verify the efficacy based on the monosaccharides contained in fucoidan. Therefore, ongoing research is necessary.

Immune checkpoint inhibitors are recognized as groundbreaking cancer treatments [25]. Immune checkpoint inhibitors block the action of immune tolerance proteins expressed on tumor cells to guide T cells to avoid attacking tumors [25,26]. In particular, CTL activation by immune checkpoint inhibitors plays a crucial role in killing [26,27]. However, it is challenging to use in patients with low expression of immune tolerance proteins, and even in patients with high expression of these proteins, there are cases in which it does not work for unknown reasons [28,29]. Accordingly, various efforts are being made to improve the efficacy of immune checkpoint inhibitors [6,30]. Attempts to enhance the effectiveness of immune checkpoint inhibitors by using adjuvants that activate immunity have shown promising results, and recent studies have focused on developing appropriate adjuvants [6,30]. Adjuvants that enhance the efficacy of immune checkpoint inhibitors focus on inducing CTL activity [6,11]. The intranasal administration of DAF potently induced the activation of helper T cells and CTLs in the mLN. This contributed sufficiently to improve the function of immune checkpoint inhibitors; therefore, it effectively inhibited the growth of lung cancer when used in combination with anti-PD-L1 antibodies. Based on results from animal experiments using mice, we plan to conduct additional studies related to human immune activity and develop immune checkpoint inhibitors as adjuvants to enhance the function of immune checkpoint inhibitors in the treatment of cancer in humans.

DCs are critical for T-cell activation [18,31]. Unlike DCs induced by in vitro differentiation, DCs in vivo induce the activity of helper T cells or CTLs depending on their subtype [17,18]. Intranasal administration of DAF potently induced the activation of cDC1 in mLN. In the case of cDC2, a similar degree of activation was observed in response to DAF or LPS, which served as positive controls. In contrast, cDC1 expressed more potent activating factors than LPS. Considering that DAF induces cDC1 activity more strongly than LPS, we expected it to induce CTL activity more strongly than LPS. However, DAF did not show stronger CTL activity than that of LPS, due to pro-inflammatory cytokines [20] and the cDC1 co-stimulator. Pro-inflammatory cytokines are used as indicators of immune activity and contribute to immune cell activation and cytotoxicity against pathogens. Among pro-inflammatory cytokines, IL-12 is an important differentiation-inducing factor for the activity of helper T cells and CTLs [32]. DAF induced the overexpression of cDC1-activated macrophages but limited the expression of IL-12. In comparison, LPS showed low expression of cDC1 activation markers, but high production levels of IL-12. Therefore, the reason DAF induced cDC1 activity more strongly than LPS while CTL activity was similar, may be due to the difference between these two factors.

Fucoidans bind to various receptors. The Toll-like receptor (TLR), scavenger receptor (SR), and Dectin-1 are representative examples [33,34]. Interestingly, these receptors are highly expressed in immune cells and are involved in immune activation and suppression. Therefore, fucoidan is thought to bind to these receptors and regulate immune cell activity. We previously confirmed that DAF activated splenic DCs in a TLR4-dependent manner [6]. Based on the complete inhibition of DC activation by DAF at TLR4, DAF is thought to have a higher binding affinity for TLR4 than for other receptors [6].

In this study, we demonstrated that DAF, which has immune-activating properties, enhances the anticancer effect of the anti-PD-L1 antibody and inhibits the growth of lung cancer. However, it is unknown whether the results derived from this study can be applied to human tumors, which are in a state such as immune-desert or immune-cold tumors, where it is difficult for immune cells to penetrate [35]. Treatment of non-small cell lung cancer with immunotherapy is complicated because it is known to be a cold tumor [36,37]. Recently, strategies for treating cold tumors have been studied to induce antigen-specific immune activity that allows T cells to infiltrate the tumors [35,38]. In this method, activated T cells, such as Chimeric Antigen Receptor T-cell (CAR-T) therapy or cancer vaccines, can react to antigens, recognize tumor cells, and exhibit higher specificity for antigens than the anticancer effect of anti-PD-L1 antibodies [35,38]. Although this study confirmed the enhancement of the anticancer effect of anti-PD-L1 antibody by DAF, we plan to conduct additional research on cancer vaccines and enhance the activity of CAR-T cells to develop strategies for treating cold tumors.

Pro-inflammatory cytokines play a direct role in the activation of immune cells and elimination of pathogens, but are also known to induce inflammatory and autoimmune diseases [39]. DAF induced significantly lower expression of pro-inflammatory cytokines but showed superior DC activation ability compared to LPS. The expression of pro-inflammatory cytokines is also an important factor in T cell differentiation and activation [40]. However, the overexpression of pro-inflammatory cytokines induces a cytokine storm, potentially leading to sepsis [41]. Although LPS has excellent immune-activating ability, it is known as an endotoxin that induces the expression of pro-inflammatory cytokines [42]. Thus, the limited secretion of pro-inflammatory cytokines by DAF may have various applications as adjuvants for immune activation.

Oral administration of fucoidan is being studied most actively. Studies on immune activation and regulation have investigated the effects of intraperitoneal and intravenous administration. This may be because the abdominal cavity and blood are rich in immune cells, allowing fucoidan to be delivered directly to these cells. Therefore, in this study, DAF was administered nasally rather than orally to confirm the lung immune activity. When DAF was administered orally, no changes in lung immunity were observed, whereas when DAF was administered nasally, DC activity in the mLN was confirmed, leading to its application in lung cancer immunotherapy.

Following intraperitoneal administration in a previous study, we investigated pulmonary immune activation following nasal administration of DAF in this study. The results suggested that DAF functions as an immune-activating adjuvant in tumor treatment. Compared with research on tumors, research on infectious diseases using immune activity is lacking. To develop vaccines or treatments for infectious diseases, it is necessary to induce specific immune activities against antigens. Therefore, we plan to verify the induction of antigen-specific immune activity using DAF and experimental antigens such as ovalbumin, and to conduct research on the development of therapeutic or preventive vaccines for infectious diseases.

## 4. Materials and Methods

### 4.1. Mice

C57BL/6 mice (18 ± 0.2 g) were purchased from Orient Bio (Gyeonggi, Republic of Korea). The mice were provided with regular food and water and housed under pathogen-free conditions. All animal experiments were approved and conducted in accordance with the guidelines of the National Research Council Guide for the Care and Use of Laboratory Animals and the Institutional Animal Care and Use Committee at Asan Medical Center (Protocol number: 2023-20-260).

### 4.2. Cell Line

LL2 cells (passage 25) were purchased from ATCC (Manassas, VA, USA) and cultured in RPMI-1640 medium supplemented with 10% fetal bovine serum, 2 mM glutamine, 100 μg/mL streptomycin, 100 U/mL penicillin, and 1 M HEPES at 37 °C in a 5% CO_2_ environment. The cells were sub-cultured every 2–3 d.

### 4.3. Reagents and Antibodies

The DAF collected along the coast of Argentine Patagonia was purchased from Elicityl (Crolles, France). The purity of the DAF was 87%. The chemical and monosaccharide composition of DAF was characterized in our previous study [6]. LPS was purchased from Sigma-Aldrich (St. Louis, MO, USA). Fluorescently labeled Brilliant Violet 605 anti-mouse CD80 (Clone no. 16-10A1, cat no. 104729), Brilliant Violet 785 anti-mouse CD11c (Clone no. N418, cat no. 117335), PE/Cyanine7 anti-mouse CD86 (clone no. GL-1, cat no. 105013), PerCP/Cyanine5.5 anti-mouse H-2Kb (clone no. AF6-88.5, cat no. 116515), APC anti-mouse CD40 (Clone no. 3/23, cat no. 124611), PerCP anti-mouse I-A/I-E (Clone no. M5/114.15.2, cat no. 107623), FITC anti-mouse IFN-γ (Clone no. XMG1.2, cat no. 505805), PE anti-mouse TNF-α (Clone no. MP6-XT22, cat no. 506307) and PE anti-mouse FoxP3 (clone no. MF-14; cat no. 126403) was purchased from BioLegend (San Diego, CA, USA).

### 4.4. Nasal Treatment Using DAF

For intranasal administration of DAF, the nape of the neck of C57BL/6 mice was held between the index and ring fingers. After holding the mouse’s head to prevent it from moving, the mice were flipped over with their tails pointing upward and received nasal inhalation of 50 mg/kg/50 μL of DAF. The drug was administered by placing a drop at the tip of the pipette as slowly as possible to prevent the drug from entering the mouth. After all drugs were administered, the mice were held upside down for approximately 2 min to ensure drug absorption.

### 4.5. DC Analysis in mLN

DAF was administered to C57BL/6 mice, and the mLN were harvested 24 h later. The mLN were ground on a glass slide and washed with cold PBS. The pellet was resuspended in cold PBS and stained with fluorescently labeled antibodies. The mLN cells were stained with lineage Abs, anti-CD8α, and anti-CD11c. The lineage antibodies used were against CD90.1, CD3, Gr-1, B220, CD49b, and TER-119. mLN cDCs were analyzed for CD11c^+^ cells. The cDC subsets were further divided based on CD8 expression as CD8-positive cDC1 and CD8-negative cDC2.

### 4.6. Enzyme-Linked Immunosorbent Assay

The secretion of pro-inflammatory cytokines was measured in the BAL after the administration of 50 mg/kg DAF and LPS. An ELISA was performed according to the manufacturer’s instructions (BioLegend, San Diego, CA, USA). Briefly, the capture antibody for the corresponding cytokine was coated onto a 96-well plate the day before analysis, and non-specific binding was blocked for 2 h with blocking buffer containing fetal bovine serum. The samples were loaded and incubated for 2 h. After washing five times, the detection antibody was added and incubated for 1 h. After washing five times, horseradish peroxidase-conjugated secondary antibody was added and incubated for 30 min. Color development was monitored by adding a substrate and analyzed using a plate reader (Hangzhou Allsheng Instruments Co., Ltd., Hangzhou, China) after adding the stop solution.

### 4.7. Intracellular Cytokine Staining

The mLN cells were incubated with 2 μM of monensin solution (BioLegend) for 2 h. Cell viability was assessed using a Zombie Violet Fixable Viability Kit (BioLegend). The cells were then stained with anti-TCR-β, anti-CD4, and anti-CD8^+^ antibodies for 20 min at 4 °C and fixed with fixation buffer (BioLegend) for 20 min. The cells were washed with Perm buffer (BioLegend) and stained with anti-IFN-γ, anti-TNF-α, anti-granzyme B, and anti-perforin Abs for 20 min. The intracellular cytokine production was analyzed using a NovoCyte flow cytometer (ACEA Biosciences Inc., San Diego, CA, USA) in TCR-β^+^CD4^+^ and TCR-β^+^CD8^+^ T cells.

### 4.8. Lung Cancer Model and Treatment

C57BL/6 mice were administered 2 × 10^5^ LL2 cells via tail vein injection. Subsequently, 50 mg/kg/50 μL of DAF and 10 mg/kg/100 μL of anti-PD-L1 antibodies were administered at 3 d intervals 7 d after tumor cell injection. DAF was administered intranasally, and anti-PD-L1 antibody was administered intraperitoneally, and the survival of the mice was monitored.

### 4.9. Hematoxylin and Eosin (H&E) Staining

The lungs were fixed on day 15 after LL2 cell injection by injecting 1 mL of 3.7% formaldehyde into the lungs and then harvested. Harvested lungs were incubated in 3.7% formaldehyde for 24 h, followed by dehydration using acetone and chloroform. The lung tissue was embedded in paraffin, sectioned into 5 μm slices, and attached to a glass slide. After drying, the sections were rehydrated with xylene and observed under a microscope after staining with hematoxylin and eosin.

### 4.10. CD4-Positive and CD8-Positive Cell Depletion

Starting on day 6 after tumor administration, anti-CD4 and anti-CD8 antibodies (both 10 mg/kg) were administered at 2 d intervals. Starting 7 d after tumor administration, 50 mg/kg DAF was intranasally administered at 3 d intervals, and the survival of the mice was observed.

### 4.11. Statistical Analysis

All results are expressed as the mean and standard error of the mean. Unless otherwise stated, the experiments were repeated twice, and six samples were analyzed (n = 6). Statistical significance was evaluated using one-way analysis of variance and calculated using Tukey’s test. Statistical significance was set at *p* < 0.05.

## 5. Conclusions

Based on the immune activity of DAF following intraperitoneal administration, the pulmonary immune activity induced by intranasal administration was verified. Nasal administration of DAF induced the activation of cDC1 and cDC2 in the mLN, as well as potent CTL activation. Nasal administration of DAF inhibited lung cancer growth, and combined administration with anti-PD-L1 showed more potent inhibition of lung cancer growth. Thus, DAF can be used as an immune-activating adjuvant that induces lung immune activity and is expected to enhance the function of immune checkpoint inhibitors in lung cancer treatment.

## Data Availability

The data discussed in this study are available upon request from the corresponding author.

## Supplementary Materials

The following supporting information can be downloaded at: https://www.mdpi.com/article/10.3390/ph18091354/s1, Figure S1: C-C chemokine receptor type 7 (CCR7) expression in mLN DCs. The numbers indicate mean fluorescence intensity; Figure S2: Co-stimulators and MHC molecules in cDC1 and cDC2; Figure S3: Expression of co-stimulators and MHC molecules in DC by DAF injection route; Figure S4: Analysis of T cell activation by DAF.

## Abbreviations

The following abbreviations are used in this manuscript:

DAF	*Durvillaea antarctica* fucoidan
PD-L1	Programmed cell death ligand-1
DC	Dendritic cell
cDC	Conventional DC
mLN	Mediastinal lymph node
CTL	Cytotoxic T lymphocyte
IFN-γ	Interferon-gamma
TNF-α	Tumor necrosis factor-alpha
MHC	Major histocompatibility complex
PD-L1	Programmed death-ligand 1
CCR7	C-C chemokine receptor type 7
LPS	Lipopolysaccharide
BAL	Bronchoalveolar lavage
IL	Interleukin
LL2	Lewis lung carcinoma 2
LN	Lymph node
PBS	Phosphate-buffered saline
