# Supplementary material for: Nasal Administration of Durvillaea antarctica Fucoidan Inhibits Lung Cancer Growth in Mice Through Immune Activation
Source: Pharmaceuticals (Basel). 2025 Sep 9;18(9):1354. doi: 10.3390/ph18091354 (PMC12472368; doi:10.3390/ph18091354)
Supplement: Supplementary file 1 [file pharmaceuticals-18-01354-s001.zip › pharmaceuticals-3790825-supplementary.pdf]

Supplementary data

Nasal administration of *Durvillaea antarctica* fucoidan inhibits lung cancer growth in mice through immune activation

Hee-Sung Kim [1], Peter C.W. Lee [2,*], Jun-O Jin [3,*]

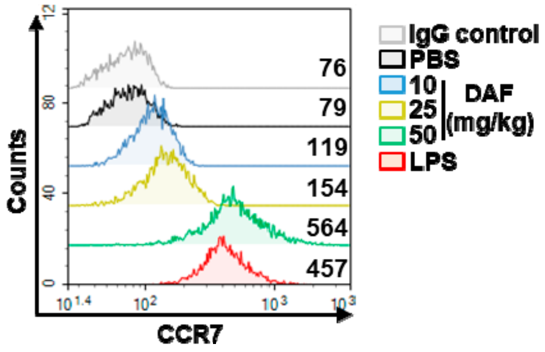

Figure S1. C-C chemokine receptor type 7 (CCR7) expression in mLN DCs. The numbers indicate mean fluorescence intensity.

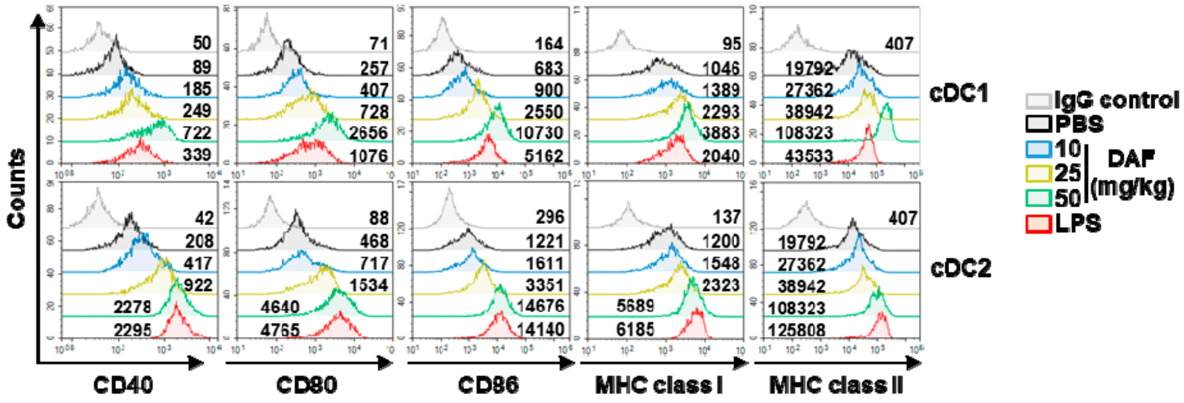

Figure S2. Co-stimulators and MHC molecules in cDC1 (upper panel) and cDC2 (lower panel). The numbers indicate mean fluorescence intensity.

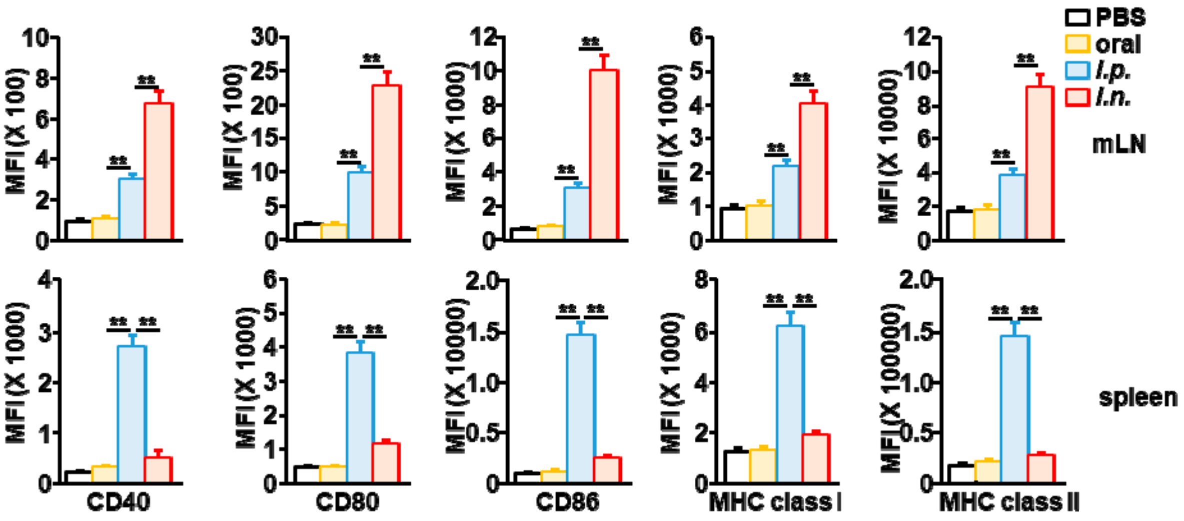

Figure S3. Expression of co-stimulators and MHC molecules in DC by DAF injection route. C57BL/6 mice were administered 50 mg/kg DAF orally (oral), intraperitoneally (*i.p.*), or intranasally (*i.n.*). Co-stimulators and MHC molecules were analyzed in mLN and splenic DCs.

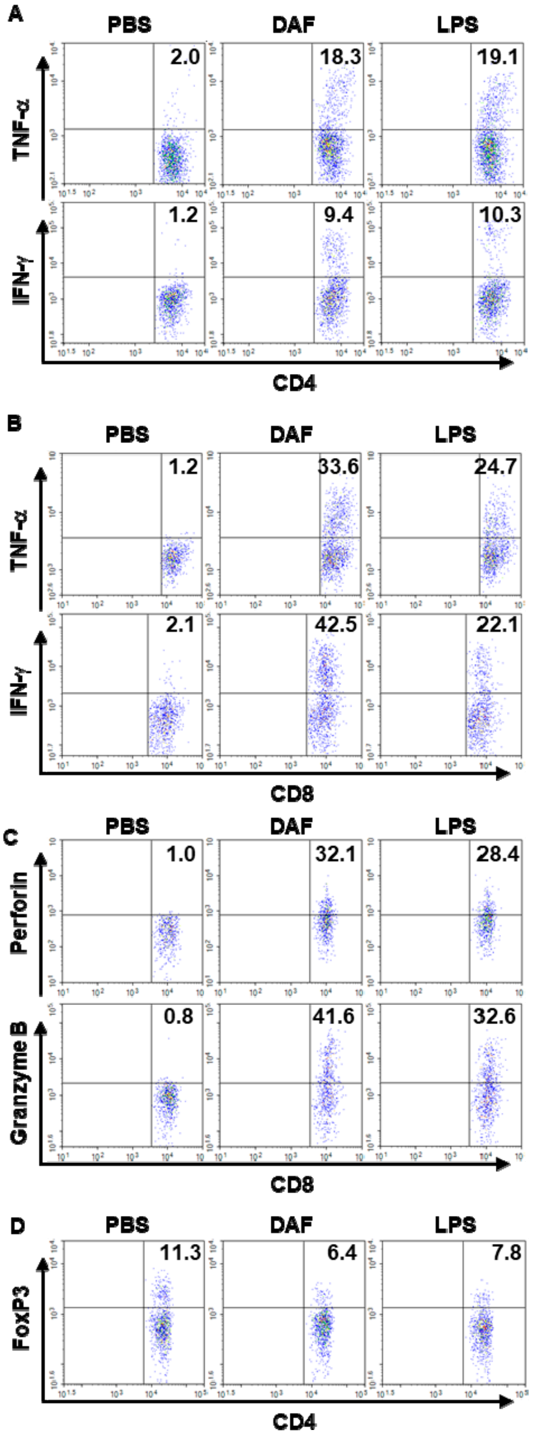

Figure S4. Analysis of T cell activation by DAF. DAF was administered to the mice, as shown in Figure 4. (A) Intracellular levels of IFN-γ and TNF-α in mLN $CD4^+$ T cells are shown (B) IFN-γ and TNF-α producing levels in mLN $CD8^+$ T cells were analyzed using a flow cytometer (C) Mean positive cells of perforin and granzyme B in mLN $CD8^+$ T cells. (D) Population of FoxP3positive cells in $CD4^+$ T cells in mLNs are shown.
